# Interactive Reference Point Procedure Based on the Conic Scalarizing Function

**DOI:** 10.1155/2014/242803

**Published:** 2014-02-27

**Authors:** Ozden Ustun

**Affiliations:** Department of Industrial Engineering, Dumlupınar University, Evliya Çelebi Campus, 43100 Kutahya, Turkey

## Abstract

In multiobjective optimization methods, multiple conflicting objectives are typically converted into a single objective optimization problem with the help of scalarizing functions. The conic scalarizing function is a general characterization of Benson proper efficient solutions of non-convex multiobjective problems in terms of saddle points of scalar Lagrangian functions. This approach preserves convexity. The conic scalarizing function, as a part of a posteriori or a priori methods, has successfully been applied to several real-life problems. In this paper, we propose a conic scalarizing function based interactive reference point procedure where the decision maker actively takes part in the solution process and directs the search according to her or his preferences. An algorithmic framework for the interactive solution of multiple objective optimization problems is presented and is utilized for solving some illustrative examples.

## 1. Introduction

A multiobjective optimization (MOP) problem can be stated as

(1) $$\begin{array}{c} \underset{x\in X}{\min}\left(f_{1}\left(x\right),\ldots ,f_{q}\left(x\right)\right), \end{array}$$

where *X* is the feasible region in decision space; *Y* is the feasible region in the objective space, where *y* ∈ *Y* if and only if there exists an *x* ∈ *X* such that *y* = (*f*_1_(*x*),…, *f*_*q*_(*x*)).

**Definition 1**

A vector *y* ∈ *Y* is an efficient point if and only if there does not exist another y* ∈ *Y* such that *y*_i_* ≤ *y*_*i*_ for all *i* and *y*_i_* < *y*_*i*_ for at least one *i*. The set of efficient points *Y*_*N*_ consists of all such efficient objective vectors. A point *x* ∈ *X* is an efficient solution if and only if its objective vector *y* = (*f*_1_(*x*),…, *f*_*q*_(*x*)) is an efficient point. The set of efficient solutions *X*_*E*_ consists of all efficient solutions in decision space.

Let *N*_*i*_ and *U*_*i*_ be the maximum and minimum values of the *i*th individual objective over the set of efficient points *Y*_*N*_. We define the maximum and minimum values of each objective over the set of efficient points *Y*_*N*_ as

(2) $$\begin{array}{c} N_{i}=\underset{y\in Y_{N}}{\max}f_{i}\left(x\right),\;\;i=1,\ldots ,q,\\ U_{i}=\underset{y\in Y_{N}}{\min}f_{i}\left(x\right),\;i=1,\ldots ,q. \end{array}$$

The vector of *N*_*i*_ values, *N*, is usually referred to as the *nadir point* while the vector of *U*_*i*_ values, *U*, is often labeled the *ideal point*.

A reference point consists of desirable values for each objective function. For decision makers (DMs), reference points are a natural way of expressing desires in solutions because DMs do not have to learn to use new, artificial concepts. Instead, objective function values are used that as such are meaningful and understandable for DMs.

The use of reference points appeared in the early development of multiple objective programming as part of the work of Charnes and Cooper [1] on goal programming. Wierzbicki [2] produced seminal research on reference point methods, including an investigation of the characteristics of various achievement functions for allowing the search for attractive efficient solutions to be controlled by reference points. These achievement functions were designed to have a significant advantage over goal programming by producing only efficient, or Pareto-optimal, solutions. Reference point methodology provides the foundation for many interactive search procedures in multiple objective programming. Over the last decades, various interactive methods and decision support systems have been developed to deal with MOPs. Among interactive approaches, methods using reference points (for the idea see, e.g., [3, 4]) have been popular (for some comparative studies, see, e.g., [5, 6]) because of their straightforward nature. Examples of methods utilizing reference points include the reference point method [4], the visual interactive approach [7], STOM [8], GUESS [9], and light beam search [10]. In addition, methods based on classification are closely related to reference point methods because a reference point can be formed once a classification has been made [11, 12]. Interactive methods enable the reduction of the computational effort and aid the DM in the decision process. In interactive methods, the set of efficient solutions is explored by a progressive articulation of the DM's preferences. This is a shared feature of all interactive methods, but there are different paradigms.

Interactive procedures are closely related to scalarization functions. Scalarization means combining different objectives into a single one such that the obtained single objective optimization problem allows finding (all) efficient (or properly efficient) solutions of the initial multiobjective problem. A variety of scalarization methods for finding efficient solutions of multiple objective programs MOPs have been developed. Some of the methods were designed specifically for linear problems and others work well only on problems with concave objective functions and a convex feasible region. However, most of the mathematical programming models of real life problems have discrete variables. Since the set of efficient solutions for problems with discrete variables is not convex, weighted sums of the objective functions do not provide a way of reaching every efficient solution. Besides the supported, there exist unsupported efficient points—points that are dominated by convex combinations of other efficient points. Conic scalarization based programs have the advantage over weighted-sums programs of being able to reach not only supported, but also unsupported efficient points [13–15]. The conic scalarization function falls into the class of achievement scalarizing functions [16, 17]. Depending on the properties of these functions, they can be used to identify (weakly, properly) efficient solutions. Most of them differ from the conic scalarizing functions by using a norm term of higher order (e.g., the Euclidean or maximum norm) in addition to the weighted-sum term. The conic scalarizing functions presented in [13, 14] guarantee a complete characterization of all properly efficient solutions.

A general characterization for the set of Benson proper efficient point was first proposed by Gasimov [13, 14]. Gasimov introduced an explicit class of increasing convex functions which serve for combining different objectives into a single one without any restrictions on objectives and constraints of the problem under consideration, such as convexity and/or boundedness. These functions are used to characterize the Benson proper efficient points of nonconvex multiobjective problems in terms of saddle points of scalar Lagrangian functions introduced in the paper [13, 14]. The conic scalarization method has been successfully applied to several multiobjective problems. Firstly, the conic scalarization method was applied by Ozdemir and Gasimov [18] to the nonconvex multiobjective faculty course-assignment problem with nine objective functions. This integrated approach consists of an analytic hierarchy process (AHP), the conic scalarization, and the modified subgradient algorithm. In the study, the weights of the objectives were calculated by using AHP. The calculated weights were used for combining the objectives by using the conic scalarization method. Ismayilova et al. [19] proposed a similar approach to solve the multiobjective faculty course-time-slot assignment problem. AHP and analytic network process (ANP) were used to determine the weights of conflicting objectives. Efficient points corresponding to the obtained weights by using AHP and ANP have been calculated and the results compared. The conic scalarization method has also been applied to the 1.5-dimensional assortment problem with two objective functions by Gasimov et al. [20]. In the study, the authors focused on the possible nonsupported or “hidden” efficient points. Since the problem under consideration involves integer variables, it becomes nonconvex. In these situations a DM may not be sure whether all the efficient points corresponding to own preferences (weights) are found by using the weighted-sum scalarization. To overcome this drawback, Gasimov et al. [20] proposed a way to obtain nonsupported efficient points based on the conic scalarization function.

Another application area of the conic scalarization method is the multiobjective portfolio optimization [17, 21]. Ehrgott et al. [17] compared multiattribute utility theory (MAUT) with the conic scalarization method. The calculated weights by using UTADIS method were used for combining the objectives in the conic scalarization method. The nonsupported or “hidden” efficient points can be obtained by using the conic scalarization method. Ustun and Kasimbeyli [21] calculated different efficient points of a multiobjective portfolio optimization problem based on combined forecasts with 11 objectives.

These approaches based on the conic scalarizing function take place either in the class of a priori methods or the class of a posteriori methods. The conic scalarization method is used as a posteriori method for the multiobjective portfolio optimization problem and the 1.5-dimensional assortment problem. A posteriori methods can also be called methods for generating efficient points. After the set of efficient points (or a part of it) has been generated, it is presented to the DM, who selects the most preferred among the alternatives. The drawbacks here are that the generation is usually computationally expensive and sometimes at least partly difficult or impossible. On the other hand, it is hard for the DM to select from among a large set of alternatives. If there are only two objective functions in a mathematical model, a set of efficient points can be generated parametrically (see, e.g., [20]). When there are more than two objectives, the problem becomes more complicated [17]. The conic scalarization method is also used as an a priori method for the nonconvex multiobjective faculty course-assignment problem and the multiobjective faculty course-time-slot assignment problem. In the a priori methods, the DM must specify the preferences, hopes, and opinions before the solution process. The main difficulty is that the DM does not necessarily know beforehand what is possible to attain in the problem and how realistic such expectations are. The existing conic scalarization approaches in the literature do not allow changes of the weights and/or reference points by the DM during the solution process according to learning. Additionally, these approaches do not support the DM in the search for more satisfactory solutions. If the DM wants to trade off among some objective function values for a current solution, she/he should tune the weights of the objective functions and/or the reference point. Unfortunately, the tuning process for obtaining more satisfactory solutions is usually computationally expensive or sometimes impossible using the existing conic scalarization approaches in the literature.

The main aim of this paper is to proposean interactive reference point procedure based on a conic scalarizing function to cope with these drawbacks to the use of the conic scalarizing function in the literature. We use the main idea of interactive classification based multiobjective optimization methods [3, 22]. The main idea of interactive classification based multiobjective optimization methods is that the DM examines the values of the objective functions calculated at a current efficient point and classifies the objective functions into different classes. This means that the DM is asked to indicate (by means of a classification) what kind of a solution would be more satisfactory than the current one [3, 11, 22]. Many scalarizing functions produce only weakly efficient solutions but such solutions are not necessarily interesting to a DM because some objective function may be improved without impairing any of the others [6]. The conic scalarization method guarantees obtaining a Benson proper efficient solution according to the multiobjective problem without solving an additional subproblem. The proposed procedure allows the DM being asked to say which objective functions have acceptable values, which should be improved, and which could be impaired. In this way, the DM has a flexible decision-making procedure to move around in the set of efficient points towards more satisfactory solutions. By using classification, we can avoid the well-known drawbacks related to expressing preference information in the form of weighting coefficients or generating the set of efficient points in the existing conic scalarization approaches.

The rest of this paper is organized as follows. We present some concepts, basic theorems, related results, and methods in Section 2. In Section 3, we introduce classification based conic scalarizing functions and the related single objective subproblems with some theoretical results for them. The conic scalarization based interactive reference point procedure is given in Section 4. Then, we apply the proposed procedure to some illustrative or comparative examples in Section 5. The paper is concluded in Section 6.

## 2. Conic Scalarizing Function, Related Results, and Methods

We briefly present some definitions and two main results from Gasimov [13].

Let *R*_+_^*q*^ = {*y* = (*y*_1_,…, *y*_*q*_) ∈ *R* ^*q*^ | *y*_*i*_ ≥ 0, *i* = 1,…, *q*}.

**Definition 2**

Let *S* be a nonempty subset of *R* ^*q*^.

- An element *s* ∈ *S* is called a Pareto minimal element of the set *S*, written as *s* ∈ min⁡⁡(*S*), if ({*s*} − *R*_+_^*q*^)∩*S* = {*s*}.
- An element *s* ∈ *S* is called a properly minimal element of *S* (in the sense of Benson), written as *s* ∈ *p* − min⁡⁡(*S*), if *s* is a Pareto minimal element of *S* and the zero element of *R* ^*q*^ is a Pareto minimal element of cl⁡(cone⁡(*S* + *R*_+_^*q*^ − {*s*})), where cl⁡ denotes the closure of the set and cone⁡(*S*): = {*δs* | *δ* ≥ 0  and  *s* ∈ *S*}.

Let *f*(*x*) = (*f*_1_(*x*),…, *f*_*q*_(*x*)), and let *f*(*X*) be the image of *X*.

**Definition 3**

$\overset{-}{x}\in X$ is called an efficient solution of (1) if $f(\overset{-}{x})\in \min(f(X))$ ; $\overset{-}{x}\in X$ is called a proper efficient point of (1) (in the sense of Benson), if $f\left(\overset{-}{x}\right)\in p-\min(f(X))$ .

Let

(3) W:={(α,w)∈R×R+q ∣ 0≤α<min⁡⁡{w1,…,wq}, wi≥0,  i=1,…,q}.

**Theorem 4 (see [13])**

Suppose that for some (*α*, *w*) ∈ *W* an element $\overset{-}{x}\in X$ is an optimal solution to the following scalar minimization problem:

(4) $$\begin{array}{c} \underset{x\in X}{\min}\left[\alpha \overset{q}{\underset{i=1}{\sum }}\left|f_{i}\left(x\right)\right|+\overset{q}{\underset{i=1}{\sum }}{w_{i}f}_{i}\left(x\right)\right]. \end{array}$$

Then, the solution $\overset{-}{x}\in X$ is a proper efficient solution of (1).

**Theorem 5 (see [13])**

Suppose that $\overset{-}{x}\in X$ is a proper efficient solution of (1). Then there exists an element (*α*, *w*) ∈ *W*, such that $\overset{-}{x}\in X$ is an optimal solution to the following scalar minimization problem:

(5) $$\begin{array}{c} \underset{x\in X}{\min}\left[\alpha \overset{q}{\underset{i=1}{\sum }}\left|f_{i}\left(x\right)-f_{i}\left(\overset{-}{x}\right)\right|+\overset{q}{\underset{i=1}{\sum }}w_{i}\left(f_{i}\left(x\right)-f_{i}\left(\overset{-}{x}\right)\right)\right]. \end{array}$$

Theorem 4 asserts that any solution of the (scalar) problem (4) is an efficient solution of the problem (1). On the other hand, Theorem 5 claims that every efficient solution $\overset{-}{x}$ of (1) can be calculated by solving a scalar problem of the form (5) for some pair (*α*, *w*) ∈ *W*. Thus, these theorems assert that the problem (1) can be scalarized in the form (4) and/or (5), and all efficient solutions of (1) can be calculated by solving scalar problems of these forms. Regarding the solutions of problem (1), note that in the case where the signs of the objective functions remain unchanged in the set of feasible solutions, the absolute values in (4) are not essential in the whole expression, and it reduces to an expression representing the weighted-sum scalarization. This situation can be overcome as follows. It is well known and easy to show that the following two problems have the same set of efficient solutions:

(6) $$\begin{array}{c} \underset{x\in X}{\min}\left(f_{1}\left(x\right),\ldots ,f_{q}\left(x\right)\right), \end{array}$$

(7) $$\begin{array}{c} \underset{x\in X}{\min}\left(f_{1}\left(x\right)-b_{1},\ldots ,f_{q}\left(x\right)-b_{q}\right), \end{array}$$

where *b*_1_,…, *b*_*q*_ are arbitrary fixed numbers. By choosing these numbers in the interior of ranges of *f*_1_,…, *f*_*q*_, respectively, we can replace the objective functions by new ones for which the conic scalarization approach will work more effectively. When the multiobjective problem under consideration is nonconvex, by taking different values for the parameter *α* and the numbers *b*_1_,…, *b*_*q*_, different efficient solutions corresponding to the same set of weights *w*_1_,…, *w*_*q*_ can be obtained by solving the scalarized version of the problem (7) as follows:

(8) $$\begin{array}{c} \underset{x\in X}{\min}\left[\alpha \overset{q}{\underset{i=1}{\sum }}\left|f_{i}\left(x\right)-b_{i}\right|+\overset{q}{\underset{i=1}{\sum }}w_{i}\left(f_{i}\left(x\right)-b_{i}\right)\right]. \end{array}$$

Additionally, the analyst must call attention to properties of the conic scalarizing function (8).

Note that the conic scalarizing function (5) for *α* = 0 or *b* ≤ *U* reduces to the weighted-sum scalarization. Therefore, nonsupported efficient solutions can only be found with *α* > 0 and *b* > *U*.

**Proposition 6**

If the reference point *b* ∈ *R* ^*q*^ satisfies one of the conditions *α* = 0 or *b* ≤ *U*, then the conic scalarizing function in (5) is equivalent to the weighted-sum scalarizing function ∑_*i*=1_^*q*^ *w*_*i*_ *f*_*i*_(*x*).

**Proof**

It is clear that the conic scalarizing function in (5) for *α* = 0 reduces to the weighted-sum scalarizing function ∑_*i*=1_^*q*^ *w*_*i*_(*f*_*i*_(*x*) − *b*_*i*_). On the other hand, if *b* ≤ *U*, then *b* ≤ *U* ≤ *f*(*x*), for *x* ∈ *X*. In this case, |*f*_*i*_(*x*) − *b*_*i*_| = *f*_*i*_(*x*) − *b*_*i*_, for all *i* = 1,…, *q*.

We obtain *α*∑_*i*=1_^*q*^|*f*_*i*_(*x*) − *b*_*i*_| + ∑_*i*=1_^*q*^ *w*_*i*_(*f*_*i*_(*x*) − *b*_*i*_) = *α*∑_*i*=1_^*q*^(*f*_*i*_(*x*) − *b*_*i*_) + ∑_*i*=1_^*q*^ *w*_*i*_(*f*_*i*_(*x*) − *b*_*i*_) = ∑_*i*=1_^*q*^(*α* + *w*_*i*_)*f*_*i*_(*x*) − ∑_*i*=1_^*q*^(*α* + *w*_*i*_)*b*_*i*_.

This is equivalent to the weighted-sum scalarizing function.

### 2.1. A Priori Method Based on the Conic Scalarizing Function

A priori methods based on the conic scalarizing function were proposed by Ozdemir and Gasimov [18] and Ismayilova et al. [19] to solve the multiobjective faculty course-assignment problems. The main steps of these methods can be given as follows.

*Step 1*. Construct a multiobjective model for a problem under consideration.

*Step 2*. Calculate the weights of the objectives using the AHP and/or the ANP proposed by Saaty [23, 24]. Choose a reference point.

*Step 3*. Synthesize the multiobjective problem by using the weights and the reference point determined at Step 2 for the conic scalarizing function to obtain a single objective problem.

*Step 4*. Solve the synthesized problem for the different values of *α*. Show the obtained efficient points to DM to obtain a final solution.

### 2.2. A Posteriori Method Based on the Conic Scalarizing Function

A posteriori methods were used to solve multiobjective portfolio optimization problems by Ehrgott et al. [17] and Ustun and Kasimbeyli [21] and the 1.5-dimensional assortment problem with two objective functions by Gasimov et al. [20]. The main steps of these approaches can be given as follows.

*Step 1*. Generate a group of well-dispersed weight vectors and choose a reference point from the interior of ranges of the objective functions. Scalarize the given problem by using the conic scalarizing function.

*Step 2*. Solve the scalarized problem for each weight vector. Show the obtained efficient points to DM for a final solution.

In the existing a posteriori methods, authors tried to reach the different nonsupported or “hidden” efficient points that cannot be calculated by the weighted-sum scalarization. To reach possible nonsupported efficient points, different weight vectors and reference points were firstly generated. The scalarized problem is solved by using these parameters. Then obtained hidden efficient points are diagrammatically illustrated in a figure. The most systematical approach among these studies is given by Gasimov et al. [20]. Gasimov et al. [20] propose the following way to obtain a group of well-dispersed weight vectors and to select a reference point in Step 1 for a biobjective programming problem.

Authors utilized formula (8) for all possible integer triples (*α*, *w*_1_, *w*_2_) ∈ *W* with *w*_1_, *w*_2_ = 1,2,…, 50, *w*_1_ + *w*_2_ = 50, and *α* = 1,2,…, min⁡{*w*_1_, *w*_2_}. The reference points initially can be arbitrarily chosen between the “relatively distant” from each other. After calculating additional efficient points, the intervals for choosing the nonzero values for reference points in the conic scalarizing function can be sequentially tightened and DM can find other nonsupported efficient solutions (if they exist) in this way, by which the authors obtained different linearly nonsupported efficient points.

## 3. Classification Based Conic Scalarizing Function and Related Subproblems

In this section, we introduce some background for the classification based conic scalarizing function to be used in this study. We use the general idea of classification in multiobjective optimization given by Miettinen [3, 22]. The idea of interactive classification based multiobjective optimization methods is that the DM examines the values of the objective functions calculated at a current efficient solution *x* ^*c*^ and classifies the objective functions into different classes. This means that the DM is asked to indicate (by means of classification) what kind of a solution would be more satisfactory than the current one. In this paper, we use up to four different classes and they are for functions *f*_*i*_ whose values

1. should be decreased (*i* ∈ *I* ^<^),
2. should be decreased down to some aspiration level (*i* ∈ *I* ^≤^),
3. are satisfactory at the moment (*i* ∈ *I* ^=^),
4. are allowed to increase up to some upper bound (*i* ∈ *I* ^≥^).

The DM is asked to specify the aspiration levels *b*_*i*_ for *i* ∈ *I* ^≤^ satisfying *b*_*i*_ ≤ *f*_*i*_(*x* ^*c*^) and the upper bounds *ε*_*i*_ for *i* ∈ *I* ^≥^ such that *ε*_*i*_ ≥ *f*_*i*_(*x* ^*c*^). The difference between the classes *I* ^<^ and *I* ^≤^ is that functions in *I* ^<^ are to be minimized as far as possible but functions in *I* ^≤^ only to the aspiration level. The idea is that with classification, the DM directs the solution process in order to find the most preferred efficient solution. That is why a classification is feasible only if at least some of the objective functions should improve and some are allowed to increase from their current levels, that is, four different classes. We can also assume that in a feasible classification, an objective function that has reached its ideal value cannot be assigned to either of the classes *I* ^<^ ∪ *I* ^≤^.

We can update the conic scalarization function by using the classification information expressed by DM. We update the conic scalarizing function by using the original objective functions and the preference information about the four different classes specified. We propose the following single objective subproblem:

(9)  minimize [α∑i|fi(x)−bi|+∑iwi(fi(x)−bi)]

(10)  subject  to fi(x)≤fi(xc) ∀i∈I<∪I≤∪I=,

(11)   fi(x)≤εi ∀i∈I≥,

(12)   fi(x)≥bi ∀i∈I≤,

(13)   x∈X.

The constraints (12) provide lower bounds for *f*_*i*_(*x*) for all *i* ∈ *I* ^≤^, because these objective functions should be decreased down to *b*_*i*_. There is another way to provide that the functions should be decreased down to *b*_*i*_ for all *i* ∈ *I* ^≤^. Weights of *f*_*i*_(*x*) for all *i* ∈ *I* ^≤^ can be taken equal to the value of parameter *α* in (8). In this way, the constraints (12) are eliminated. This result can be shown as follows:

(14) α∑i|fi(x)−bi|+∑iwi(fi(x)−bi)  ={∑i(α+wi)(fi(x)−bi),if  fi(x)≥bi,∑i(wi−α)(fi(x)−bi),if  fi(x)≤bi.  

If *w*_*i*_ is taken equal to *α* in (14), we obtain

(15) α∑i|fi(x)−bi|+∑iwi(fi(x)−bi)  ={∑i2α(fi(x)−bi),if  fi(x)≥bi,0,if  fi(x)≤bi.  

Thus it provides that the functions should be decreased down to *b*_*i*_ for all *i* ∈ *I* ^≤^. But the taking of *w*_*i*_ = *α* for any *i* contradicts the theoretical results of the conic scalarization such that (*α*, *w*) ∈ *W*. To eliminate this contradiction, we take the related weighting values to be *w*_*i*_ = *α* + *ρ* > 0, where *ρ* is a small scalar for all *i* ∈ *I* ^≥^ ∪ *I* ^≤^ ∪ *I* ^=^. On the other hand, the values *f*_*i*_(*x*) for *i* ∈ *I* ^≥^ should be allowed to increase up to *ε*_*i*_. The DM can specify the aspiration levels *b*_*i*_ = *ε*_*i*_ for *i* ∈ *I* ^≥^ for this requirement. Additionally, the values *f*_*i*_(*x*) for *i* ∈ *I* ^=^ for a current efficient solution *x* ^*c*^ are required to remain at its current value, *f*_*i*_(*x* ^*c*^).

We have also used a denominator to scale each term in the objective function of (8) to be of a similar magnitude. This aims to capture the preferences of the DM better. Note that by the definitions of ideal and nadir point, we have *N*_*i*_ − *U*_*i*_ > 0 for all *i* = 1,…, *q*.

After these modifications of the subproblem (9)–(13), we obtain the following subproblem:

(16)   minimize [α∑i|fi(x)−bi|Ni−Ui+∑iwi(fi(x)−bi)Ni−Ui]

(17)   subject  to fi(x)≤fi(xc) ∀i∈I<∪I≤  ∪I=,

(18)   fi(x)≤εi ∀i∈I≥,

(19)   x∈X,

where *w*_*i*_ = *α* + *ρ* > 0, where *ρ* is a small scalar for all *i* ∈ *I* ^≥^ ∪ *I* ^≤^ ∪ *I* ^=^, *b*_*i*_ = *ε*_*i*_ for all *i* ∈ *I* ^≥^, and *b*_*i*_ = *f*_*i*_(*x* ^*c*^) for all *i* ∈ *I* ^=^.

The subproblem (16)–(19) combines the conic scalarization method with the *ε*-constraint method. It is a hybrid method which considers both of DM's aspiration levels and reservation levels by the classification point of view.

**Theorem 7**

Let (*α*, *w*) ∈ *W*. A feasible solution x* ∈ *X* is an optimal solution of the subproblem (16)–(19) if and only if x* ∈ *X* is a proper efficient solution of (1).

**Proof**

There exists a feasible solution of the subproblem (16)–(19), because *x* ^*c*^ is a current efficient solution, and *ε*_*i*_ ≥ *f*_*i*_(*x* ^*c*^) for *i* ∈ *I* ^≥^. Let x* ∈ *X* be efficient solution of the subproblem (16)–(19). Then there is no *x* ∈ *X* such that *f*(*x*) ≤ *f*(x*). Thus any feasible solution of the subproblem (16)–(19) satisfies *f*(*x*) = *f*(x*) and is an optimal solution.

While (*α*, *w*) ∈ *W*, we have *α* ≥ 0, *w*_*i*_ > *α*, and *N*_*i*_ − *U*_*i*_ > 0 for all *i*. Let x* ∈ *X* be an optimal solution of the subproblem (16)–(19). Then there is no *x* ∈ *X* such that *f*(*x*) ≤ *f*(x*) and

(20) α∑i|fi(x)−bi|Ni−Ui+∑iwi(fi(x)−bi)Ni−Ui  ≤α∑i|fi(x∗)−bi|Ni−Ui+∑iwi(fi(x∗)−bi)Ni−Ui

(obtained from the result of Theorems 4 and 5). This means that x* is a proper efficient solution of (1).

## 4. Conic Scalarizing Function Based Interactive Reference Point Procedure

After summarizing the necessary mathematical background, we can introduce the conic scalarization based interactive reference point procedure.

Before the solution process starts, some information is given to the DM about the problem. If possible, the ideal point and the nadir point are presented to illustrate the ranges of the set of efficient points *Y*_*N*_. Another possibility is to minimize and maximize the objective functions individually in the feasible region. Thus if the feasible region is bounded, then the DM is informed about the best and worst values of the objective functions. If calculating the ideal or nadir points is difficult in practice and/or any element of these points is infinite, the denominators *N*_*i*_ − *U*_*i*_ in the objective function (16) can be removed for all *i*. The theoretical result given in Theorem 7 is valid in this situation.

The main steps of the conic scalarization based interactive reference point procedure are the following.

*Step 1* (initialization). Set *k* = 0. Convert the maximization objectives into minimization ones by multiplying the objective functions by −1. Present information about the problem to the DM. If possible, calculate the ideal and nadir points. Present them to the DM. Ask the DM to specify a finite reference point *b* ^*k*^ ∈ *R* ^*q*^.

*Step 2* (weights determination). Ask the DM to specify weights *w* ^*k*^ ∈ *R* ^*p*^ of the objective functions for *i* ∈ *I* ^<^. If *k* = 0, then all objective functions are in the class *I* ^<^ and set *p* = *q*. The analytic hierarchy process (AHP) is proposed to the DM for determining the weights of the objective functions *w* ^*k*^ ∈ *R* ^*p*^ [23] in this step. Ask the DM to construct a pairwise comparison matrix of the relative contributions of each objective function by using Saaty's 9-point scale as follows:

(21) f1(x)f2(x)⋯fp(x)f1(x)1a12⋯a1pf2(x)1a121⋯a2p⋮⋮⋮1⋮fp(x)1a1p1a2p⋯1

where *a*_*ij*_ is the relative importance of objective function *i* compared to objective function *j*.

Compute the consistency ratio (CR) of the pairwise comparison matrix. Accept the matrix if CR is less than 0.10. Otherwise, check the judgments on the relative importance of the objective functions to eliminate the inconsistency until CR is less than 0.10.

Compute the priorities for each objective function. Use these priorities as the weights of objective functions *w* ^*k*^ = (*w*_1_^*k*^,…, *w*_*p*_^*k*^) after normalization.

*Step 3* (most preferred solution). If *k* = 0, then all objective functions are in the class *I* ^<^ and use *f*(*x* ^*k*^) = *N*, where *N* is nadir point (or the worst values of the objective functions in the feasible region) and *b* ^*k*^ ∈ *R* ^*q*^, by satisfying *b* ^*k*^ < *f*(*x* ^*k*^).

Choose the number of intervals *m* for sampling *α* values. Calculate *α*_*t*_ = *λt*, *t* = 0,1,…, *m* − 1, where *λ* = min⁡⁡{*w*_1_^*k*^,…, *w*_*p*_^*k*^}/*m*. Solve the subproblem for each *α*_*t*_ value:

(22)  minimize  [αt∑i|fi(x)−bik|Ni−Ui+∑iwik(fi(x)−bik)Ni−Ui],

(23)  subject  to   fi(x)≤fi(xk) ∀i∈I<∪I≤  ∪I=,

(24)      fi(x)≤εi ∀i∈I≥,

(25)      x∈X,

where *w*_*i*_^*k*^ = *α*_*t*_ + *ρ* > 0, where *ρ* is a small scalar for all *i* ∈ *I* ^≥^ ∪ *I* ^≤^ ∪ *I* ^=^, *b*_*i*_^*k*^ = *ε*_*i*_ for all *i* ∈ *I* ^≥^, and *b*_*i*_^*k*^ = *f*_*i*_(*x* ^*k*^) for all *i* ∈ *I* ^=^.

Present the solutions of the subproblems according to the values of *α*_*t*_ to the DM. Ask the DM to determine the most preferred solution among the calculated solutions for different values of *α*. Set *α* = *α*_*l*_, where the *l*th efficient point *y* ^*k*^ is the most preferred by the DM. Let *x* ^*k*^ be an efficient solution related to *y* ^*k*^.

*Step 4* (alternative solutions). Calculate a number, *q*, of other efficient solutions by solving the subproblem (22)–(25) for *α* = *α*_*l*_ with perturbed reference points

(26) $$\begin{array}{c} y^{k}\left(i\right)=b^{k}+d^{k}e^{i}, \end{array}$$

where *d* ^*k*^ = ||*b* ^*k*^ − *y* ^*k*^|| and *e* ^*i*^ is the *i*th unit vector for *i* = 1,…, *q*.

*Step 5* (decision). Present the alternatives to the DM. If the DM finds one of the *q* + 1 efficient points satisfactory, then the DM chooses it as the final solution. Otherwise, set *k* = *k* + 1. Ask the DM to classify the objective functions into four different classes such as *I* ^=^, *I* ^<^, *I* ^≤^, and *I* ^≥^. Let *p* be the number of objectives in the class *I* ^<^. Also ask the DM to specify aspiration levels *b*_*i*_^*k*^ for *i* ∈ *I* ^<^ ∪ *I* ^≤^ satisfying *b*_*i*_^*k*^ < *f*_*i*_(*x* ^*k*−1^) and upper bounds *ε*_*i*_ such that *ε*_*i*_ ≥ *f*_*i*_(*x* ^*k*−1^) for *i* ∈ *I* ^≥^. Go to Step 2.

It is clear that if *m* increases, then the DM can select a more sensible efficient solution in Step 3. But large values of *m* require more computational time. In this study the parameters of the conic scalarization function are interactively determined by the DM. The weights of the objectives are calculated by using AHP at each iteration of the procedure. The value of the *α* parameter is chosen by the DM after solving *m* subproblems. The proposed procedure must solve *m* + *q* subproblems at each iteration. Thus at each iteration, the DM evaluates *m* + *q* efficient solutions (some of these can be the same according to the shape of the efficient frontier) and learns whether the goals are realistic or not. The advantage of perturbing the reference point in Step 4 is that the DM gets a better conception about possible solutions. If the reference point is far from the set of efficient points, the DM gets a wider description of the set of efficient points, and if the reference point is near the set of efficient points, then a finer description of the set of efficient points is given.

## 5. Illustrative Examples

We now examine the conic scalarization based interactive reference point procedure in the context of some nonconvex MOP problems. Utility functions were used to simulate DM preferences with different weights of objective functions. Thus the solution quality of a reference point and an efficient point can be evaluated and compared by using DM's utility function. The mathematical models in this study are solved by using the Lingo 11.0 solver. There is no limitation for the number of variables, integer variables, and constraints. Lingo 11.0 can solve linear, integer, mixed integer, and nonlinear programming models [25]. We also compute the priority for each objective function and consistency ratios of pairwise comparison matrices by using the trial version of Expert Choice 11.5. The trial version of Expert Choice 11.5 can be downloaded at the URL (http://expertchoice.com/). An Intel Core 2 Duo 2.27 GHz Notebook PC with WINDOWS 7 operating system on one processor is used in the computations.

### 5.1. Examples

**Example 1**

Let us consider the multiobjective mixed integer linear programming model for supplier selection and order allocation which follows [26]:

(27)  min⁡f1(x,z)=12.22x1+14x2+12.4x3+10x4 +100z1+75z2+120z3+80z4,

(28)  min⁡f2(x)=0.003x1+0.003x2 +0.004x3+0.004x4, −0.23x3−0.23x4,

(29)  min⁡f3(x)=−0.29x1−0.25x2 −0.23x3−0.23x4,

(30)  subject  to x1+x2+x3+x4≥10000,

(31)   x1−4000z1≤0,

(32)   x2−2500z2≤0,

(33)   x3−3500z3≤0,

(34)   x4−3500z4≤0,

(35)   xi≥0, i=1,…,4,

(36)   zi=0  or  1 integer, i=1,…,4.

The objective functions *f*_1_(*x*, *z*), *f*_2_(*x*), and *f*_3_(*x*) are, respectively, the sum of the material cost and the order cost, the expected number of defective products, and thetotal value of purchasing. We assume that the DM wants to join the interactive decision-making process and that DM's implicit utility function is UT(*f*(*x*, *z*)) = (−*f*_1_(*x*, *z*) − *f*_2_(*x*) − *f*_3_(*x*))/3. Let *X* be the feasible set determined by the constraints (30)–(36). The main steps of the conic scalarization based interactive reference point procedure are simulated as follows.

*First Iteration*

*Step 1* (initialization). Set *k* = 0. The analyst or computer program calculates the ideal point and nadir point as *U* = (115180,33.5, −3395) and *N* = (165655,47.5, −2540), respectively. Details about these points are given in Table 1.

The calculated ideal and nadir points are presented to the DM, who specifies a reference point *b* ^0^ = (*U* + *N*)/2 = (138917.5,40.5, −2967.5) with UT(*b* ^0^) = −44876.9.

*Step 2* (weights determination). Because *k* = 0, all objective functions are in the class *I* ^<^ and set *p* = 3. The DM constructs a pairwise comparison matrix of the relative contribution of each objective function by using Saaty's 9-point scale as follows:

(37) f1(x)f2(x)f3(x)f1(x)111f2(x)111f3(x)111

Although CR = 0 is less than 0.10, the pairwise comparison matrix is consistent. The calculated priorities of the objective functions are 0.33, 0.33, and 0.33, respectively. Use these priorities as the weights of the objective functions *w* ^0^ = (*w*_1_^0^, *w*_2_^0^, *w*_3_^0^) = (0.33, 0.33, 0.33).

*Step 3* (most preferred solution). Because *k* = 0, all objective functions are in the class *I* ^<^. Use *b* ^0^ ∈ *R* ^3^ satisfying *b* ^0^ < *f*(*x* ^0^) and *f*(*x* ^0^) = *N* = (162655,47.5, −2540), where *N* is the nadir point.

Let *m* be 10. Solve the following subproblem for *α*_*t*_ = *λt*, *t* = 0,1,…, 9, where *λ* = min⁡⁡{0.33, 0.33,0.33}/10 = 0.033:

(38)   minimize  [αt∑i|fi(x)−bi0|Ni−Ui+∑iwi0(fi(x)−bi0)Ni−Ui], subject  to  f1(x,z)≤f1(x0,z0)=162655,   f2(x)≤f2(x0)=47.5,   f3(x)≤f3(x0)=−2540,   x∈X.

The solutions of the subproblems according to the values of *t* are given in Table 2.

Ask the DM to determine the most preferred solution among the calculated solutions for the different values of *t*. Set *α* = *α*_3_ = 0.1, where the 3rd efficient point *y* ^0^ = (119135,33.5, −2590) is the most preferred by the DM. The efficient solution related to *y* ^0^ is *x* ^0^ = (4000,2500,0, 3500).

*Step 4* (alternative solutions). The analyst calculates three other efficient solutions by solving the subproblem given in Step 3 with perturbed reference points *y* ^0^(*i*) = *b* ^0^ + *d* ^0^ *e* ^*i*^, where *d* ^0^ = ||*b* ^0^ − *y* ^0^|| = 19786.1 and *e* ^*i*^ is the *i*th unit vector for *i* = 1,2, 3.

The efficient points according to the perturbed reference points are given in Table 3.

*Step 5* (decision). The efficient points obtained in Table 3 are the same with *y* ^0^. The DM wants to continue the process for a more attractive solution. Set *k* = *k* + 1. The DM classifies the objective functions into two different classes. There are two objective functions *f*_1_(*x*, *z*) and *f*_3_(*x*) in the class *I* ^<^. The objective function *f*_2_(*x*) is in the class *I* ^≥^. The DM specifies aspiration levels *b*_1_^1^ = 118000 and *b*_3_^1^ = −2600 for *i* ∈ *I* ^<^ satisfying *b*_*i*_^1^ < *f*_*i*_(*x* ^0^) and the upper bounds *ε*_2_ = 38 ≥ *f*_*i*_(*x* ^0^) = 33.5 for *i* ∈ *I* ^≥^. Go to Step 2.

*Second Iteration*

*Step 2* (weights determination). The DM constructs a pairwise comparison matrix of the relative contribution on each objective function by using Saaty's 9-point scale as follows:

(39) f1(x)f3(x)f1(x)11f3(x)11

Although CR = 0 is less than 0.10, the pairwise comparison matrix is consistent. The calculated priorities of objective functions are 0.5 and 0.5, respectively. Use these priorities as the weights of objective functions *w* ^1^ = (*w*_1_^1^, *w*_3_^1^) = (0.5,0.5).

*Step 3* (most preferred solution). Solve the subproblem for *α*_*t*_ = *λt*, *t* = 0,1,…, 9, where *λ* = min⁡⁡{0.5,0.5}/10 = 0.05:

(40)  minimize [αt∑i|fi(x)−bi1|Ni−Ui+∑iwi1(fi(x)−bi1)Ni−Ui], subject  to f1(x,z)≤f1(x0,z0)=119135,  f2(x)≤ε2=38,  f3(x)≤f3(x0)=−2590,  x∈X,

where *w*_2_ = *α*_*t*_ + *ρ* > 0, *ρ* = 0.0001, and *b*_2_^1^ = 38.

The solutions of the subproblems according to the values of *t* are given in Table 4.

Ask the DM to determine the most preferred solution among the calculated solutions for different values of *t*. Set *α* = *α*_9_ = 0.45, where the 9th efficient point *y* ^1^ = (118414.8,37.04, −2600) is selected as the most preferred efficient point by the DM. The efficient solution related to *y* ^1^ is *x* ^1^ = (4000,0, 2760.87,3500).

*Step 4* (alternative solutions). The analyst calculates three other efficient points by solving the subproblem in Step 3 for *α* = *α*_9_ = 0.45 with perturbed reference points *y* ^1^(*i*) = *b* ^1^ + *d* ^1^ *e* ^*i*^, where *d* ^1^ = ||*b* ^1^ − *y* ^1^|| = 414.8 and *e* ^*i*^ is the *i*th unit vector for *i* = 1,2, 3.

The obtained efficient points according to the perturbed reference points are given in Table 5.

*Step 5* (decision). The DM finds one of the four efficient points satisfactory and then chooses *y* ^1^(3) = (118000,36.91, −2592.31) as the final solution.

**Example 2**

Let us consider a mixed integer nonlinear multiobjective problem with 5 objectives, 40 binary variables, 40 positive variables corresponding to assets, and 3 constraints [17, 27]. Let *n* be the number of available assets, *x*_*i*_ and *r*_*i*_ the fraction of the available capital invested in asset *i* and the expected return of asset *i* for *i* = 1,…, *n*, and *σ*_*ij*_ the covariance of the returns of assets *i* and *j*. For asset *i* ∈ {1,…, *n*}, let *r*_*i*_^12^ be the 12-month performance, *r*_*i*_^36^ the 36-month (long-term) performance (expected return), *d*_*i*_ ≥ 0 the relative annual dividend, and *s*_*i*_ ∈ {1,…, *n*} the number of stars assigned to asset *i* (one star indicates relatively poor performance of assets and five stars indicate very good performance). We have used a dataset of *n* = 40 investment funds from Standard and Poor's 1999 database. Consider the following five objective functions.

*f*_01_(*x*) = ∑_*i*=1_^*n*^ *r*_*i*_^12^ *x*_*i*_ is the 12-month performance. This objective function is a measure for the short-term expected return.

*f*_02_(*x*) = ∑_*i*=1_^*n*^ *r*_*i*_^36^ *x*_*i*_, the 3-year performance, is a measure for the long-term expected return.

*f*_03_(*x*) = ∑_*i*=1_^*n*^ *d*_*i*_ *x*_*i*_ represents the relative annual dividend of a portfolio.

*f*_04_(*x*) = ∑_*i*=1_^*n*^ *s*_*i*_ *x*_*i*_ is the average star ranking of portfolio risk. Standard and Poor's Fund Service evaluates the performance of most investment funds contained in their data based on an annual basis which results in a performance ranking.

*f*_05_(*x*) = ∑_*i*=1_^*n*^∑_*j*=1_^*n*^ *σ*_*ij*_ *x*_*i*_ *x*_*j*_ is the usual variance measure of portfolio risk.

Let *z*_*i*_, *i* = 1,…, *n*, denote binary variables with *z*_*i*_ = 1 if and only if asset *i* is contained in the portfolio. It is assumed that exactly *k* assets must be held in the portfolio. Thus, we consider the cardinality-constrained (*k* = 10) portfolio optimization problem of size *n* = 40 in Example 2:

(41)  min⁡   f1(x)=−∑i=140ri12xi,

(42)  min⁡   f2(x)=−∑i=140ri36xi,

(43)  min⁡   f3(x)=−∑i=140dixi,

(44)  min⁡   f4(x)  =−∑i=140sixi,

(45)  min⁡   f5(x)=∑i=1n∑j=1nσijxixj,

(46)  subject  to ∑i=140xi=1,

(47)   ∑i=140zi=10,

(48)   0.05zi≤xi≤0.3zi, i=1,…,40,

(49)   xi≥0, i=1,…,40,

(50)   zi=0  or  1 integer, i=1,…,40.

This problem is an extended version of the Markowitz mean-variance model.

We assume that DM's implicit utility function is UT(*f*(*x*)) = −0.163*f*_1_(*x*) − 0.003*f*_2_(*x*) − 0.032*f*_3_(*x*) − 0.535*f*_4_(*x*) − 0.268*f*_5_(*x*). Let *X* be the feasible set determined by the constraints (46)–(50). The main steps of the conic scalarization based interactive reference point procedure are simulated for Example 2 as follows.

*First Iteration*

*Step 1* (initialization). Set *k* = 0. The analyst or computer program calculates the ideal point *U* and the nadir point *N*. The ideal and nadir solutions are calculated as *U* = (−280.02, −278.80, −5.94, −5.00,0.39) and *N* = (−17.64, −43.09, −0.29, −2.40,0.79). Details about these points are given in Table 6.

The calculated ideal and nadir points are presented to the DM, who specifies a reference point *b* ^0^ = (*U* + *N*)/2 = (−148.83, −160.95, −3.12, −3.7,0.59) with the utility value UT(*b* ^0^) = 26.66.

*Step 2* (weights determination). Because *k* = 0, all objective functions are in the class *I* ^<^ and *p* = 5. The DM constructs a pairwise comparison matrix of the relative contribution of each objective function by using Saaty's 9-point scale as follows:

(51) f1(x)f2(x)f3(x)f4(x)f5(x)f1(x)1991312f2(x)19111919f3(x)19111919f4(x)39912f5(x)29921

Although CR = 0.04 is less than 0.10, the pairwise comparison matrix is consistent. The calculated priorities of the objective functions are 0.210, 0.033, 0.033, 0.432, and 0.293, respectively. Use these priorities as the weights of objective functions *w* ^0^ = (*w*_1_^0^,…, *w*_5_^0^) = (0.210, 0.033, 0.033, 0.432, 0.293).

*Step 3* (most preferred solution). Because *k* = 0, all objective functions are in the class *I* ^<^. Use *b* ^0^ ∈ *R* ^5^ satisfying *b* ^0^ < *f*(*x* ^0^) and *f*(*x* ^0^) = *N* = (−17.64, −43.09, −0.29, −2.40,0.79). Let *m* be 3. Solve the subproblem for *α*_*t*_ = *λt*, *t* = 0,1, 2, where *λ* = (min⁡⁡{0.210, 0.033, 0.033, 0.432, 0.293})/3 = 0.011:

(52)  minimize [αt∑i|fi(x)−bi0|Ni−Ui+∑iwi0(fi(x)−bi0)Ni−Ui], subject  to f1(x)≤f1(x0)=−17.64,  f2(x)≤f2(x0)=−43.09,  f3(x)≤f3(x0)=−0.29,  f4(x)≤f4(x0)=−2.40,  f5(x)≤f5(x0)=0.79,  x∈X.

The solutions of the subproblems according to the values of *t* are given in Table 7.

Ask the DM to determine the most preferred solution among the calculated solutions for different values of *α*. Set *α* = *α*_0_ = 0, where the first efficient solution *y* ^0^ = (−193.55, −195.92, −0.76, −4.61,0.52) in Table 7 is the most preferred by the DM.

*Step 4* (alternative solutions). The analyst calculates five other efficient solutions by solving the subproblem given in Step 3 with perturbed reference points, *y* ^0^(*i*) = *b* ^0^ + *d* ^0^ *e* ^*i*^, where *d* ^0^ = ||*b* ^0^ − *y* ^0^|| = 56.83 and *e* ^*i*^ is the *i*th unit vector for *i* = 1,…, 5.

The efficient points obtained are given according to the perturbed reference points in Table 8.

*Step 5* (decision). The efficient points obtained are the same as with *y* ^0^ given at Step 4. The DM finds *y* ^0^ satisfactory and then chooses*y* ^0^ = (−193.55, −195.92, −0.76, −4.61,0.52) as the final solution.

### 5.2. Comparative Example

**Example 3**

Let us consider the nonconvex multiobjective programming problem as follows:

(53)  max⁡   f1(x)=x1, max⁡   f2(x)=x2, subject  to x12+x22≥1,  x1≤1,  x2≤1,  x1,x2≥0.

It is clear that the feasible region is bounded and nonconvex. The efficient set is *Y*_*N*_ = {(*y*_1_, *y*_2_) ∈ *R* ^2^ | *y*_1_^2^ + *y*_2_^2^ = 1, *y*_1_, *y*_2_ ≥ 0}. Therefore, the ideal and nadir points exist. The ideal point *U* = (1,1) and the nadir point *N* = (0,0) of the problem can be found by optimizing objective functions individually. In this example, a multiplicative-additive utility function was used to simulate DM preferences. We assume that DM's implicit multiplicative-additive utility function is UT(*f*(*x*)) = 0.4*f*_1_(*x*) + 0.3*f*_2_(*x*) + 0.3*f*_1_(*x*)*f*_2_(*x*). DM's optimal solution can be obtained by maximizing *U*(*x*) under the constraints of Example 3. The optimal solution is x* = (0.7513,0.6600) with the utility value UT(*f*(x*)) = 0.6473.

We compare the proposed interactive method with the existing approaches based on the conic scalarization by using the example. The existing approaches and the proposed method are employed to maximize DM's implicit utility function.

**Case 1**

Firstly, we applied the a priori method given in Section 2.1 to Example 3. The objective functions are transformed to the minimization form by multiplying −1.

*Step 1.* We consider the multiobjective problem given in Example 3.

*Step 2.* The weights of the objectives are calculated by using the AHP. We assume that DM constructs a pairwise comparison matrix of the relative contribution on each objective function by using Saaty's 9-point scale as follows:

(54) f1(x)f2(x)f1(x)143f2(x)341

Although CR = 0 is less than 0.10, the pairwise comparison matrix is consistent. The calculated priorities of objective functions are 0.571 and 0.429, respectively. These priorities are used as the weights of objective functions *w* = (0.571,0.429). Let DM's reference point be *b* = (−0.5, −0.5).

*Step 3.* We synthesized the multiobjective problem by using the weights and the reference point determined at Step 2. The obtained single objective problem due to the value of *α* can be given as follows:

(55) min⁡z=α(|−x1+0.5|+|−x2+0.5|)+0.571(−x1+0.5)+0.429(−x1+0.5),subject  to   x12+x22≥1, x1≤1, x2≤1, x1,x2≥0.

*Step 4.* The synthesized problems were solved for the different values of *α* such as 0.1, 0.2, 0.3, and 0.4. The obtained efficient points are given in Table 9.

It is clear that DM chooses the efficient solution (0.7995, 0.6007) with the highest utility value 0.6441 among the efficient solutions given in Table 9.

**Case 2**

We applied the a posteriori method given in Section 2.2 to Example 3.

*Step 1.* The weight vectors are generated for all possible integer triples (*α*, *w*_1_, *w*_2_) ∈ *W* with *w*_1_, *w*_2_ = 1,2,…, 50, *w*_1_ + *w*_2_ = 50, and *α* = 1,2,…, min⁡{*w*_1_, *w*_2_}. Thus we obtain the 650 number of the weight vectors. Let DM's reference point be *b* = (−0.5, −0.5).

*Step 2.* The scalarized problem is solved for each weight vector. The highest utility value among the 650 number of the obtained efficient points is 0.6450 related to DM's final solution (0.7071, 0.7071). The final solution can be obtained for the triples (*α*, *w*_1_, *w*_2_) = {(*α*, 25,25), where *α* = 2,…, 25} by using Lingo 11.0.

**Case 3 (the proposed interactive procedure)**

The objective functions are transformed to the minimization form by multiplying −1.

*First Iteration*

*Step 1* (initialization). Set *k* = 0. The analyst or computer program calculates the ideal point and nadir point as *U* = (−1, −1) and *N* = (0,0), respectively. The calculated ideal and nadir points are presented to the DM, who specifies a reference point *b* ^0^ = (−0.5, −0.5) with UT(−*b* ^0^) = 0.425.

*Step 2* (weights determination). Because *k* = 0, all objective functions are in the class *I* ^<^ and set *p* = 2. The DM constructs a pairwise comparison matrix of the relative contribution of each objective function as follows:

(56) f1(x)f2(x)f1(x)143f2(x)341

Although CR = 0 is less than 0.10, the pairwise comparison matrix is consistent. The calculated priorities of the objective functions are 0.571 and 0.429, respectively. Use these priorities as the weights of the objective functions *w* ^0^ = (*w*_1_^0^, *w*_2_^0^) = (0.571,0.429).

*Step 3* (most preferred solution). Because *k* = 0, all objective functions are in the class *I* ^<^. Use *b* ^0^ ∈ *R* ^2^ satisfying *b* ^0^ < −*f*(*x* ^0^) and −*f*(*x* ^0^) = *N* = (0,0), where *N* is the nadir point.

Let *m* be 5. Solve the following subproblem for *α*_*t*_ = *λt*, *t* = 0,1, 2,3, 4, where *λ* = min⁡⁡{0.571, 0.429}/5 = 0.0858:

(57)  min⁡ αt(|−x1+0.5|+|−x2+0.5|)  +0.571(−x1+0.5)+0.429(−x1+0.5), s.t.   −x1≤−f1(x0)=0,    −x2≤−f2(x0)=0,    x12+x22≥1,    x1≤1,    x2≤1,    x1,x2≥0.

The solutions of the subproblems according to the values of *t* are given in Table 10.

Ask the DM to determine the most preferred solution among the calculated solutions for the different values of *t*. Set *α* = *α*_1_ = 0, where the first efficient point *y* ^0^ = (0.7995,0.6007) is the most preferred by the DM.

*Step 4* (alternative solutions). The analyst calculates two other efficient solutions by solving the subproblem given in Step 3 with perturbed reference points *y* ^0^(*i*) = *b* ^0^ + *d* ^0^ *e* ^*i*^, where *d* ^0^ = ||*b* ^0^ − *y* ^0^|| = 0.3160 and *e* ^*i*^ is the *i*th unit vector for *i* = 1,2.

The efficient points according to the perturbed reference points are given in Table 11.

*Step 5* (decision). The efficient points obtained in Table 11 are the same with *y* ^0^. The DM wants to continue the process for a more attractive solution. Set *k* = *k* + 1. The DM classifies the objective functions into two different classes. There is the second objective function *f*_2_(*x*) in the class *I* ^<^. The objective function *f*_1_(*x*) is in the class *I* ^≥^. The DM specifies aspiration levels *b*_2_^1^ = − 0.62 for *i* ∈ *I* ^<^ satisfying *b*_*i*_^1^ < *f*_*i*_(*x* ^0^) and the upper bounds *ε*_1_ = −0.75 ≥ *f*_2_(*x* ^0^) = −0.7995 for *i* ∈ *I* ^≥^. Go to Step 2.

*Second Iteration*

*Step 2* (weights determination). The DM constructs a pairwise comparison matrix of the relative contribution on each objective function by using Saaty's 9-point scale as follows:

(58) f2(x)f2(x)1

Although CR = 0 is less than 0.10, the pairwise comparison matrix is consistent. The calculated priority of the objective function is 1. Use this priority as the weight of the objective function *w* ^1^ = (*w*_2_^1^) = (1).

*Step 3* (most preferred solution). Solve the subproblem for *α*_*t*_ = *λt*, *t* = 0,1, 2,3, 4, where *λ* = (min⁡⁡{1})/5 = 0.2:

(59)  minimize [αt∑i|fi(x)−bi1|Ni−Ui+∑iwi1(fi(x)−bi1)Ni−Ui], subject  to −x1≤ε1=−0.75,  −x2≤−f2(x0)=−0.6007,  x∈X,

where *w*_2_ = *α*_*t*_ + *ρ* > 0, *ρ* = 0.0001, and *b*_1_^1^ = −0.75.

The solutions of the subproblems according to the values of *t* are given in Table 12.

Ask the DM to determine the most preferred solution among the calculated solutions for different values of *t*. Set *α* = *α*_0_ = 0, where the first efficient point *y* ^1^ = (0.75,0.6614) is selected as the most preferred efficient point by the DM.

*Step 4* (alternative solutions). The analyst calculates two other efficient points by solving the subproblem in Step 3 for *α* = 0 with perturbed reference points, *y* ^1^(*i*) = *b* ^1^ + *d* ^1^ *e* ^*i*^, where *d* ^1^ = ||*b* ^1^ − *y* ^1^|| = 0.0414 and *e* ^*i*^ is the *i*th unit vector for *i* = 1,2.

The obtained efficient points according to the perturbed reference points are given in Table 13.

*Step 5* (decision). The DM finds one of the two efficient points as satisfactory, and then DM chooses *y* ^1^(2) = (0.75,0.6614) as the final efficient point.

The results obtained by using the a priori method, the a posteriori method, and the proposed method are summarized in Table 14.

As it can be seen in Table 14, the highest utility value among the methods is obtained by using the proposed method. The proposed method allowed DM to reach the more satisfactory solution. It is clear that the existing approaches are either computationally expensive or not flexible for DM's learning process. In the a posteriori method, 650 efficient points should be compared by DM for a simple biobjective problem. On the other hand, although the more satisfactory efficient solution exists in the feasible region for Example 3, the a priori method causes DM to have to select the less satisfactory efficient solution.

## 6. Conclusions

In this study, a novel interactive approach called the conicscalarizing function based interactive reference point procedure is proposed. The proposed procedure aims to eliminate the drawbacks in current practice of the use of the conic scalarization based methods. This procedure allows a flexible multiobjective decision-making process to select a more preferred efficient solution for the DM. The basic technique of the proposed method is the subproblem (16)–(19) which combines the conic scalarization method with the *ε*-constraint method. It is a hybrid method which considers both of DM's aspiration levels and reservation levels by the classification point of view. DMs direct the proposed procedure by using four different classes to obtain more satisfactory solutions. In this way, the proposed procedure allows tradeoffs among the objectives' values by classifying. The theoretical background of the proposed method is given in detail. The proposed method was applied to illustrative examples. It is also compared with the existing methods according to the multiplicative-additive utility function by using the comparative example. The highest utility value among the methods is obtained by using the proposed method.

Further directions of the study can be given as follows: from the theoretical point of view, the proposed algorithm can be combined with different optimization techniques for effectively solving nonconvex subproblems. Also, different sampling techniques can be adapted to obtain different efficient solution sets. The main difficulty about the use of the conic scalarization technique is the tuning of the value of the *α* parameter according to the preferences of the DM. In this study, we proposed a sampling method related to *m* values. Different search methods can be used for this purpose. For example, a kind of interval reduction technique can be used. From the practical point of view, the proposed algorithm can be applied to different test problems and compared with other interactive procedures. The weights of the objectives can be calculated by using the analytic network process at each iteration of the procedure. Fuzzy numbers could be used to represent uncertain parameters of the procedure. The proposed procedure could also be used to develop software with decision support systems methodology.

## Figures and Tables

**Table 1:** The ideal and nadir points for Example 1.

Number		*f*_1_(*x*, *z*)	*f*_2_(*x*)	*f*_3_(*x*)	UT(*f*(*x*, *z*))	*x*_1_	*x*_2_	*x*_3_	*x*_4_
1	*U*_1_	115180	36	−2540	−37558.67	4000	0	2500	3500
2	*U*_2_	119135	33.5	−2590	−38859.5	4000	2500	0	3500
3	*U*_3_	162655	47.5	−3395	−53102.5	4000	2500	3500	3500
4	*U*	115180	33.5	−3395	−37272.83	—	—	—	—
5	*N*	162655	47.5	−2540	−53387.5	—	—	—	—

**Table 2:** The solutions of the subproblems according to different values of *t*.

*t*	*f*_1_(*x*, *z*)	*f*_2_(*x*)	*f*_3_(*x*)	UT(*f*(*x*, *z*))	*x*_1_	*x*_2_	*x*_3_	*x*_4_
0	119135	33.5	−2590	−38859.5	4000	2500	0	3500
1	119135	33.5	−2590	−38859.5	4000	2500	0	3500
2	119135	33.5	−2590	−38859.5	4000	2500	0	3500
3	119135	33.5	−2590	−38859.5	4000	2500	0	3500
4	138917.5	39.843	−2954.7	−45334.21	4000	2500	1585.69	3500
5	138917.5	39.843	−2954.7	−45334.21	4000	2500	1585.69	3500
6	138917.5	39.843	−2954.7	−45334.21	4000	2500	1585.69	3500
7	138917.5	39.843	−2954.7	−45334.21	4000	2500	1585.69	3500
8	138917.5	39.843	−2954.7	−45334.21	4000	2500	1585.69	3500
9	138917.5	40.5	−2958.9	−45333.03	4000	2066.49	2075.133	3500

**Table 3:** The efficient points obtained at the first iteration according to the perturbed reference points.

*i*	*y* ^0^(*i*)			*f*_1_(*x*, *z*)	*f*_2_(*x*)	*f*_3_(*x*)	UT(*f*(*x*, *z*))
1	158703.6	40.5	−2967.5	119135	33.5	−2590	−38859.5
2	138917.5	19826.6	−2967.5	119135	33.5	−2590	−38859.5
3	138917.5	40.5	16818.5	119135	33.5	−2590	−38859.5

**Table 4:** The solutions of the subproblems according to the values of *t* at the second iteration.

*t*	*f*_1_(*x*, *z*)	*f*_2_(*x*)	*f*_3_(*x*)	UT(*f*(*x*, *z*))	*x*_1_	*x*_2_	*x*_3_	*x*_4_
0	119135	37.28	−2613.34	−38852.97	4000	0	2818.95	3500
1	118414.8	37.04	−2600	−38617.28	4000	0	2760.87	3500
2	118414.8	37.04	−2600	−38617.28	4000	0	2760.87	3500
3	118414.8	37.04	−2600	−38617.28	4000	0	2760.87	3500
4	118414.8	37.04	−2600	−38617.28	4000	0	2760.87	3500
5	118414.8	37.04	−2600	−38617.28	4000	0	2760.87	3500
6	118414.8	37.04	−2600	−38617.28	4000	0	2760.87	3500
7	118414.8	37.04	−2600	−38617.28	4000	0	2760.87	3500
8	118414.8	37.04	−2600	−38617.28	4000	0	2760.87	3500
9	118414.8	37.04	−2600	−38617.28	4000	0	2760.87	3500

**Table 5:** The efficient points obtained at the second iteration according to the perturbed reference points.

*i*	*y* ^1^(*i*)			*f*_1_(*x*, *z*)	*f*_2_(*x*)	*f*_3_(*x*)	UT(*f*(*x*, *z*))
1	118414.8	38	−2600	118414.8	37.04	−2600	−38617.28
2	118000	452.8	−2600	118414.8	37.04	−2600	−38617.28
3	118000	38	−2185.2	118000	36.91	−2592.31	−38481.53

**Table 6:** The ideal and nadir points for Example 2.

Number		*f*_1_(*x*)	*f*_2_(*x*)	*f*_3_(*x*)	*f*_4_(*x*)	*f*_5_(*x*)	UT(*f*(*x*))
1	*U*_1_	−280.02	−240.80	−0.29	−4.15	0.73	48.40
2	*U*_2_	−243.00	−278.80	−0.35	−4.25	0.67	42.55
3	*U*_3_	−17.64	−43.09	−5.94	−2.40	0.79	4.27
4	*U*_4_	−126.17	−142.96	−0.43	−5.00	0.74	23.48
5	*U*_5_	−97.98	−127.38	−1.41	−3.11	0.39	17.96
6	*U*	−280.02	−278.80	−5.94	−5.00	0.39	49.24
7	*N*	−17.64	−43.09	−0.29	−2.40	0.79	4.09

**Table 7:** The solutions of the subproblems according to different values of *t* for Example 2.

*t*	*f*_1_(*x*)	*f*_2_(*x*)	*f*_3_(*x*)	*f*_4_(*x*)	*f*_5_(*x*)	UT(*f*(*x*))
0	−193.55	−195.92	−0.76	−4.61	0.52	34.49
1	−186.63	−190.21	−0.85	−4.64	0.52	33.36
2	−179.29	−184.11	−0.94	−4.66	0.52	32.16

**Table 8:** The obtained efficient points according to the perturbed reference points in Example 2.

*i*	*y* ^0^(*i*)					*f*_1_(*x*)	*f*_2_(*x*)	*f*_3_(*x*)	*f*_4_(*x*)	*f*_5_(*x*)	UT(*f*(*x*))
1	−92	−160.95	−3.12	−3.7	0.59	−193.55	−195.92	−0.76	−4.61	0.52	34.49
2	−148.83	−104.12	−3.12	−3.7	0.59	−193.55	−195.92	−0.76	−4.61	0.52	34.49
3	−148.83	−160.95	53.71	−3.7	0.59	−193.55	−195.92	−0.76	−4.61	0.52	34.49
4	−148.83	−160.95	−3.12	53.13	0.59	−193.55	−195.92	−0.76	−4.61	0.52	34.49
5	−148.83	−160.95	−3.12	−3.7	57.42	−193.55	−195.92	−0.76	−4.61	0.52	34.49

**Table 9:** The efficient points of the subproblems according to different values of *α* for Example 3.

*α*	0	0.1	0.2	0.3	0.4
*f*_1_(x*)	0.7995	0.8198	0.851	0.866	0.866
*f*_2_(x*)	0.6007	0.5726	0.525	0.5	0.5
UT(*f*(x*))	0.6441	0.6406	0.6320	0.6263	0.6263

**Table 10:** The efficient points of the subproblems according to different values of *α* for Example 3.

*α*	0	0.0858	0.1716	0.2574	0.3442
*f*_1_(x*)	0.7995	0.8164	0.8406	0.866	0.866
*f*_2_(x*)	0.6007	0.5775	0.5417	0.5	0.5
UT(*f*(x*))	0.6441	0.6412	0.6353	0.6263	0.6263

**Table 11:** The efficient points obtained at the first iteration according to the perturbed reference points.

*i*	*y* ^0^(*i*)		*f*_1_(*x*)	*f*_2_(*x*)	UT(*f*(*x*))
1	−0.159	−0.5	0.7995	0.6007	0.6441
2	−0.5	−0.159	0.7995	0.6007	0.6441

**Table 12:** The efficient points of the subproblems according to different values of *α* for Example 3.

*α*	0	0.2	0.4	0.6	0.8
*f*_1_ *f*_1_(x*)	0.75	0.75	0.75	0.75	0.75
*f*_2_(x*)	0.6614	0.6614	0.6614	0.6614	0.6614
UT(*f*(x*))	0.64725	0.64725	0.64725	0.64725	0.64725

**Table 13:** The efficient points obtained at the first iteration according to the perturbed reference points.

*i*	*y* ^1^(*i*)		*f*_1_(*x*)	*f*_2_(*x*)	UT(*f*(*x*))
1	−0.7086	−0.62	0.7086	0.7056	0.6451
2	−0.75	−0.5786	0.75	0.6614	0.64725

**Table 14:** Comparison of the results of the a priori method, the a posteriori method, and the proposed method.

Criteria/methods	The a priori method	The a posteriori method	The proposed method
Number of the solved subproblems	1	650	14
*f*(x*) = (*f*_1_(x*), *f*_2_(x*))	(0.7995, 0.6007)	(0.7071, 0.7071)	(0.75, 0.6614)
UT(*f*(x*))	0.6441	0.6450	0.64725
